# Variation in care for inpatients with avoidant restrictive food intake disorder leads to development of a novel inpatient clinical pathway to standardize care

**DOI:** 10.1186/s40337-024-01018-8

**Published:** 2024-05-23

**Authors:** Elana M. Bern, Carly E. Milliren, Kevin K. Tsang, Lisa A. Mancini, Julia K. Carmody, Marina G. Gearhart, Olivia Eldredge, Chase Samsel, McGreggor Crowley, Tracy K. Richmond

**Affiliations:** 1 Division of Gastroenterology, Hepatology and Nutrition, Boston Children’s Hospital, 333 Longwood Avenue, 5th Floor, Boston, MA 02115 USA; 2 Department of Pediatrics, Harvard Medical School, Boston, MA USA; 3 Department of Psychiatry and Behavioral Sciences, Boston Children’s Hospital, Boston, MA USA; 4 Department of Psychiatry, Harvard Medical School, Boston, MA USA; 5 Institutional Centers for Clinical and Translational Research, Boston Children’s Hospital, Boston, MA USA; 6 Division of Adolescent/Young Adult Medicine, Boston Children’s Hospital, Boston, MA USA

**Keywords:** ARFID, Inpatient care, Clinical pathway

## Abstract

**Introduction:**

There is limited evidence to guide management of patients with avoidant restrictive food intake disorder (ARFID) admitted for medical stabilization. We describe variations in inpatient care which led to the development of a multidisciplinary inpatient clinical pathway (ICP) to provide standardized management and examine differences after the ICP was implemented.

**Methods:**

A retrospective review of patients with ARFID admitted to Adolescent Medicine, Gastroenterology, and General Pediatrics at a single academic center was conducted. We compare hospital utilization and use of consulting services during the pre-ICP (2015–2017) and post-ICP (2018–2020) periods.

**Results:**

110 patients were admitted with ARFID (n = 57 pre- vs. n = 53 post-ICP). Most presented with moderate/severe malnutrition (63% pre vs. 81% post; p = 0.11) and co-morbid anxiety and/or depression (74% pre vs. 92% post; p = 0.01). There was some variation in use of enteral tube feeding by service in both periods (p = 0.76 and p = 0.38, respectively), although overall use was consistent between periods (46% pre vs. 58% post; p = 0.18). Pre-ICP, use of the restrictive eating disorder protocol differed across services (p < 0.001), with only AM using it. Overall, utilization of the restrictive eating disorder protocol decreased from 16% pre-ICP to 2% post-ICP (p = 0.02). There was variation by service in psychiatry/psychology (range 82–100% by service; p = 0.09) and social work consultations (range 17–71% by service; p = 0.001) during the pre-ICP period, though variation was reduced in the post-ICP period (p = 0.99 and p = 0.05, respectively). Implementation of the ICP led to improvements in these consultative services, with all patients in the post-ICP period receiving psychiatry/psychology consultation (p = 0.05) and an increase in social work consults from 44 to 64% (p = 0.03). Nutrition consults were consistently utilized in both periods (98% pre vs. 100% post; p = 0.33).

**Conclusion:**

The ICP was developed to standardize inpatient medical stabilization for patients with ARFID. In this single center study, implementation of the ICP increased standardized care for inpatients with ARFID with variation in care reduced: there were improvements in the use of consulting services and a reduction in the use of the restrictive eating disorder protocol. The ICP demonstrates the potential to further standardize and improve care over time.

## Abstract (plain language summary)

There is limited evidence to guide management of children and adolescents with Avoidant Restrictive Food Intake Disorder (ARFID) admitted for medical stabilization. The study describes the variation in inpatient care for ARFID, which led to the development of a multidisciplinary standardized inpatient clinical pathway (ICP). The ICP centers the experience of the patient and family with an emphasis on biopsychosocial support. Implementation of the ICP increased standardized care for inpatients with ARFID with variation in care reduced: There were improvements in the use of psychiatry/psychology and social work consulting services and a reduction in the use of the restrictive eating disorder protocol. Future research is needed to better understand the impact of the inpatient clinical pathway to improve care over time.

## Introduction

Avoidant Restrictive Food Intake Disorder (ARFID), a relatively new diagnosis, was added to the 5th edition of the Diagnostic and Statistical Manual of Mental Disorders (DSM-5) in 2013 [1]. ARFID is an eating or feeding disorder characterized by poor growth, malnutrition, reliance on nutritional supplements, and/or psychosocial impairment, in the absence of body image disturbance [2]. Children and adolescents with ARFID often present with severe medical and nutritional consequences (e.g., short stature, pubertal delay, nutrient deficiencies, low bone density) due to their restricted diets [3, 4]. In addition to physical complications, co-morbid psychiatric concerns, such as anxiety and depression, are common [5]. The codification of the ARFID diagnosis in the DSM-5 in 2013 and subsequent inclusion in the International Classification of Disease Tenth Revision (ICD-10) in 2018 [1, 6], introduced a new diagnostic grouping and associated need to develop effective and standardized approaches to care.

Given the relative newness of the diagnosis and the complexity in presentation of patients with this disorder, significant variation in care is expected. Patients with ARFID may be cared for by a variety of inpatient care teams including Adolescent/Young Adult Medicine (AM), Gastroenterology (GI), or General Pediatrics/ Hospitalist (GP) services, likely contributing to the variability in care approaches. Nutritional rehabilitation approaches also vary (e.g., in the use of restrictive eating disorder protocols or regular use of nasogastric tube feeds and may vary by admitting service) [7, 8]. ARFID-specific care protocols are lacking [9, 10].

To address the lack of existing guidance for inpatient care for patients with ARFID, we first examined the variability in care by admitting service (i.e., AM, GI, GP) prior to development of a standardized inpatient clinical pathway (ICP). We then created a standardized ICP using Quality Improvement (QI) methods. After implementation of the ICP, we examined changes in care among inpatients with ARFID post-ICP compared to before initiation of the ICP (pre-ICP). Examining differences in care was exploratory in nature, given the small cohort size. We examined clinical process measures (use of the restrictive eating disorder protocol, subspecialty consultations, use of enteral tube feeding, diagnostic endoscopic evaluation) and clinical outcomes (change in BMI z-score during hospitalization) and differences in hospital utilization (length of stay, 30-day readmissions).

## Methods

### Aims 1 and 3: Comparing care by admitting service and time period (pre- v. post-ICP implementation)

We retrospectively extracted electronic medical record (EMR) data for patients with ARFID admitted for medical stabilization from January 1, 2015–December 31, 2017 (pre-ICP) and January 1, 2018–February 29, 2020 (post-ICP) at a stand-alone, academic children’s hospital in New England. The Boston Children’s Hospital (BCH) Institutional Review Board approved this retrospective study and waived patient consent for the study.

The EMR was queried utilizing an internal tool for searching the admitting and discharge notes. Parameters were developed to retrospectively identify all patients, 4 to 21 years of age, with a first medical admission for ARFID. Admitting diagnosis of ARFID was confirmed by one of three trained reviewers based on the DSM-5 criteria for ARFID [1] and confirmation of the admitting diagnosis of ARFID in the EMR psychology consult note, when available. Our sample was limited to those admitted to the 3 admitting services most commonly caring for patients with ARFID: AM, GI, or GP. We excluded admissions to services other than AM, GI, or GP (n = 3 pre-ICP and n = 7 post-ICP) and patients admitted or discharged to the inpatient Psychiatry service (n = 5 pre-ICP and n = 13 post-ICP).

We extracted additional sociodemographic and clinical data from the EMR via our hospital’s data warehouse. We examined sociodemographic variables (gender, age, race/ethnicity, insurance payor, approximation of the actual distance traveled from home to the hospital), co-morbid medical and psychiatric diagnoses based on billed ICD-10 diagnosis codes, anthropometrics (weight, height) upon admission, length of stay (LOS), all-cause medical readmissions within 30 days (excluding psychiatric), and the admitting and discharging inpatient team (AM, GI, GP). Additional chart review was performed to extract the following information: use of enteral tube feeding (naso-gastric tube, naso-jejunal tube, or gastrostomy tube), performance and results of endoscopy, request for consultative assessment by psychiatry/psychology consultation service (PCS), nutrition or social work, and use of the existing restrictive eating disorder (RED) protocol that had been developed primarily for patients with anorexia nervosa.

### Statistical analysis

We report median (interquartile range; IQR) for continuous variables and frequency (percent) for categorical variables. We compared sociodemographic factors, clinical characteristics, and hospital utilization overall between admitting services pre-ICP and then conducted similar comparisons between the pre- and post-ICP periods using Kruskal–Wallis for continuous variables and chi-square tests for categorical variables. All analyses were performed in SAS (v9.4; Cary, NC) at an alpha-level of 0.05.

### Aim 2: Creation of a standardized inpatient clinical pathway (ICP)

With the support of an internal pilot grant, a multidisciplinary team of individuals with experience treating patients with ARFID was assembled, including specialists in Adolescent/Young Adult Medicine, Gastroenterology, Psychology, Psychiatry, Nutrition, Social Work, and Nursing. The multidisciplinary team met monthly in 2016 to develop and refine an ARFID-specific ICP. Evidence-based guidelines were used when available and supplemented with expert opinion otherwise. The ICP was developed to be tailored based on clinical (e.g., degree of malnutrition) and developmental status (e.g., age, diagnosis of autism spectrum disorder). Feedback from multi-disciplinary clinicians was incorporated via regular, iterative quality improvement steps. From initiation of the evaluation process through formulation and development of the feeding plan, the goal was to clearly define the feeding problem, reduce associated distress and address underlying triggers or co-morbid conditions while providing a framework for the team and family to work together to operationalize the treatment approach. The ultimate goal was to have a successful transition out of the hospital, either to a program or to home.

## Results

### Patient characteristics

We included a total of N = 110 admissions for patients with ARFID during the study period: n = 57 during the period pre-ICP implementation and n = 53 post-ICP was implemented. Our cohort ranged in age from 4 to 21 years. In the pre-ICP period, 24 (42%) patients were admitted to AM, 17 (30%) GI, and 16 (28%) GP compared to 25 (47%) AM, 21 (40%) GI, and 7 (13%) GP in the post-ICP period with no significant difference in volume by service between periods (p = 0.15).

### Aim 1: Differences in sociodemographic and clinical characteristics and clinical and utilization outcomes by admitting service pre-ICP

Patient sociodemographic characteristics by admitting service are presented in Table 1. Patients admitted to the AM service were older on average (p < 0.001) and were more likely to have private insurance than those admitted to the GI or GP services (p = 0.03). There were no significant differences in the weight status, co-morbid medical or psychiatric disorders by admitting service (Table 2) in the pre-ICP cohort. More than half of patients were underweight (56% overall) and co-morbid depression (18%) and anxiety (26% with generalized, 70% with other anxiety disorders) were common. Hospital utilization varied in the pre-ICP cohort (Table 3), with patients admitted to the AM service being more likely to be treated with the restrictive eating disorder protocol (p < 0.001) and less likely to have social work consulted (p = 0.001) compared to patients admitted to GI or GP. There were no differences in length of stay, readmission rates, or change in BMI by admitting service pre-ICP.

**Table 1:** Sociodemographic characteristics for ARFID inpatient admissions by admitting service before and after ICP implementation (N = 110 admissions; n = 57 pre-ICP and n = 53 post-ICP)

	Pre-ICP (n = 57)					Post-ICP (n = 53)					p-value^b^
	Median (IQR) or n (%)				p-value^a^	Median (IQR) or n (%)				p-value^a^	
	Overall (n = 57)	Admitting Service				Overall (n = 53)	Admitting Service				
		AM (n = 24)	GI (n = 17)	GP (n = 16)			AM (n = 25)	GI (n = 21)	GP (n = 7)		
Admit Age (years)	12.7 (4.5)	14.3 (5.9)	12.7 (4.6)	10.3 (3.3)	< 0.001	15.3 (5.8)	16.9 (3.6)	10.8 (6.4)	15.3 (4.4)	< 0.001	0.08
Admit Age Category					0.003					< 0.001	0.04
10 years or younger	16 (28%)	0 (0%)	7 (41%)	9 (56%)		11 (21%)	0 (0%)	11 (52%)	0 (0%)		
11 – 14 years	24 (42%)	14 (58%)	5 (29%)	5 (31%)		14 (26%)	7 (28%)	5 (24%)	2 (29%)		
15 – 17 years	9 (16%)	4 (17%)	4 (24%)	1 (6%)		21 (40%)	12 (48%)	4 (19%)	5 (71%)		
18 years or older	8 (14%)	6 (25%)	1 (6%)	1 (6%)		7 (13%)	6 (24%)	1 (5%)	0 (0%)		
Female Gender	39 (68%)	20 (83%)	11 (65%)	8 (50%)	0.08	29 (55%)	17 (68%)	6 (29%)	6 (86%)	0.006	0.14
Race					0.72					0.45	0.68
White	41 (72%)	17 (68%)	12 (71%)	12 (75%)		37 (70%)	18 (72%)	14 (67%)	5 (71%)		
Black/African-American	5 (9%)	1 (4%)	2 (12%)	2 (13%)		2 (4%)	0 (0%)	1 (5%)	1 (14%)		
Asian or Pacific Islander	1 (2%)	1 (4%)	0 (0%)	0 (0%)		3 (6%)	2 (8%)	0 (0%)	1 (14%)		
Another race	8 (14%)	3 (13%)	3 (18%)	2 (13%)		8 (15%)	4 (16%)	4 (19%)	0 (0%)		
Declined/Unknown	2 (3%)	2 (8%)	0 (0%)	0 (0%)		3 (6%)	1 (4%)	2 (10%)	0 (0%)		
Ethnicity					0.64					0.35	0.14
Non-Hispanic	46 (81%)	19 (79%)	15 (88%)	12 (75%)		40 (75%)	21 (84%)	14 (67%)	5 (71%)		
Hispanic	6 (10%)	2 (8%)	2 (12%)	2 (13%)		2 (4%)	0 (0%)	1 (5%)	1 (14%)		
Declined/Unknown	5 (9%)	3 (13%)	0 (0%)	2 (13%)		11 (21%)	4 (16%)	6 (29%)	1 (14%)		
Private Insurance	42 (74%)	22 (92%)	10 (59%)	10 (63%)	0.03	36 (68%)	17 (68%)	15 (71%)	4 (57%)	0.85	0.51
Distance traveled					0.03					0.60	0.98
< 10 miles	15 (26%)	1 (4%)	6 (35%)	8 (50%)		14 (26%)	8 (32%)	4 (19%)	2 (29%)		
10 – 24 miles	19 (33%)	11 (46%)	4 (24%)	4 (25%)		17 (32%)	8 (32%)	8 (38%)	1 (14%)		
25 – 49 miles	13 (23%)	7 (29%)	3 (18%)	3 (19%)		14 (26%)	5 (20%)	6 (29%)	3 (43%)		
50 – 99 miles	5 (9%)	4 (17%)	1 (6%)	0 (0%)		5 (9%)	2 (8%)	3 (14%)	0 (0%)		
≥ 100 miles	5 (9%)	1 (4%)	3 (18%)	1 (6%)		3 (6%)	2 (8%)	0 (0%)	1 (14%)		

*ARFID* Avoidant Restrictive Food Intake Disorder, *IQR* interquartile range, *AM* Adolescent Medicine, *GI* Gastroenterology, *GP* General Pediatrics

^a^p-value testing for difference by admitting service within each period. Admitting and discharging service were the same

^b^p-value testing for difference overall between pre and post-ICP periods

**Table 2:** Weight status at admission and co-morbid diagnoses characteristics for ARFID inpatient admissions by admitting service before and after ICP implementation (N = 110 admissions; n = 57 pre-ICP and n = 53 post-ICP)

	Pre-ICP (n = 57)					Post-ICP (n = 53)					p-value^b^
	Median (IQR) or n (%)				p-value^a^	Median (IQR) or n (%)				p-value^a^	
	Overall (n = 57)	Admitting Service				Overall (N = 53)	Admitting Service				
		AM (n = 24)	GI (n = 17)	GP (n = 16)			AM (n = 25)	GI (n = 21)	GP (n = 7)		
*Weight Status at Admission*											
BMI z-score	− 1.7 (1.7)	− 2.1 (1.6)	− 1.4 (1.8)	− 1.7 (1.8)	0.72	− 2.1 (2.3)	− 3.3 (2.0)	− 2.0 (2.2)	− 1.0 (2.8)	0.02	0.44
BMI Percentile	4.6 (11.6)	1.8 (9.1)	7.5 (14.0)	4.8 (11.5)	0.72	1.8 (15.9)	0.05 (5.2)	2.3 (16.3)	16.0 (73.9)	0.02	0.44
BMI Percentile Category^c^					0.33					0.05	0.62
Underweight (< 5th)	32 (56%)	15 (63%)	8 (47%)	9 (56%)		34 (64%)	18 (72%)	14 (67%)	2 (29%)		
Normal/healthy weight (5th–84.9th)	23 (40%)	7 (29%)	9 (53%)	7 (44%)		18 (34%)	7 (28%)	7 (33%)	4 (57%)		
Overweight or Obese (≥ 85th)	2 (4%)	2 (8%)	0 (0%)	0 (0%)		1 (2%)	0 (0%)	0 (0%)	1 (14%)		
Degree of malnutrition^d^					0.20					0.15	0.11
Severe	24 (42%)	10 (42%)	8 (47%)	6 (38%)		34 (64%)	20 (80%)	12 (57%)	2 (29%)		
Moderate	12 (21%)	9 (38%)	1 (6%)	2 (13%)		9 (17%)	2 (8%)	4 (19%)	3 (43%)		
Mild	16 (28%)	4 (17%)	6 (35%)	6 (38%)		7 (13%)	3 (12%)	3 (14%)	1 (14%)		
None	5 (9%)	1 (4%)	2 (12%)	2 (13%)		3 (6%)	0 (0%)	2 (10%)	1 (14%)		
*Co-morbid Diagnoses*											
*Medical/GI*											
Inflammatory Bowel Disease	1 (2%)	0 (0%)	1 (6%)	0 (0%)	0.30	0 (0%)	0 (0%)	0 (0%)	0 (0%)	0.99	0.99
Celiac disease	2 (4%)	0 (0%)	2 (12%)	0 (0%)	0.09	1 (2%)	1 (4%)	0 (0%)	0 (0%)	0.57	0.99
Eosinophilic Esophagitis	0 (0%)	0 (0%)	0 (0%)	0 (0%)	0.99	1 (2%)	0 (0%)	1 (5%)	0 (0%)	0.46	0.48
*Psychiatric*											
Generalized Anxiety Disorder	15 (26%)	8 (33%)	3 (18%)	4 (25%)	0.53	20 (38%)	10 (40%)	15 (71%)	3 (43%)	0.38	0.20
Other anxiety disorder	40 (70%)	17 (71%)	13 (76%)	10 (63%)	0.68	46 (87%)	20 (80%)	19 (90%)	7 (100%)	0.31	0.04
Depressive Disorder	10 (18%)	7 (29%)	2 (12%)	1 (6%)	0.13	25 (47%)	18 (72%)	1 (5%)	6 (86%)	< 0.001	0.001
*Neurodevelopmental*											
ADHD	8 (14%)	3 (13%)	2 (12%)	3 (19%)	0.81	13 (25%)	3 (12%)	9 (43%)	1 (14%)	0.04	0.16
Autism Spectrum Disorder	4 (7%)	0 (0%)	1 (6%)	3 (19%)	0.07	3 (6%)	1 (4%)	2 (10%)	0 (0%)	0.57	0.99

*ARFID* Avoidant Restrictive Food Intake Disorder, *IQR* Interquartile Range, *AM* Adolescent Medicine, *GI* Gastroenterology, *GP* General Pediatrics, *BMI* Body Mass Index, *ADHD* Attention Deficit Hyperactivity Disorder

^a^p-value testing for difference by admitting service within each period. Admitting and discharging service were the same

^b^p-value testing for difference overall between pre and post-ICP periods

^c^BMI z-score, percentile, and classification obtained from admitting Nutrition note; typically first morning weight of admission, when available. Self-reported/family reported weight loss not utilized for classification

^d^Malnutrition was defined based on BMI z-score and/or weight loss upon admission as per ASPEN definition [11]

**Table 3:** Change in weight and hospital utilization by admitting service before and after ICP implementation for ARFID inpatient admissions (N = 110 admissions; n = 57 pre-ICP and n = 53 post-ICP)

	Pre-ICP (n = 57)					Post-ICP (n = 53)					p-value^b^
	Median (IQR) or n (%)				p-value^a^	Median (IQR) or n (%)				p-value^a^	
	Overall (n = 57)	Admitting Service				Overall (N = 53)	Admitting Service				
		AM (n = 24)	GI (n = 17)	GP (n = 16)			AM (n = 25)	GI (n = 21)	GP (n = 7)		
Change in BMI z-score^c^	0.29 (0.68)	0.34 (0.86)	0.40 (0.73)	0.28 (0.59)	0.70	0.26 (0.67)	0.27 (0.74)	0.30 (0.74)	0.05 (0.23)	0.64	0.92
Change in BMI percentile^c^	0.37 (3.9)	0.35 (2.8)	0.55 (8.9)	0.24 (4.7)	0.91	0.43 (3.2)	0.33 (2.9)	0.53 (6.1)	1.30 (6.6)	0.81	0.78
Length of stay (days)	7.0 (7.4)	7.1 (7.5)	6.2 (7.1)	6.2 (7.5)	0.49	6.2 (6.7)	9.0 (5.8)	5.8 (4.7)	3.2 (12.6)	0.20	0.71
LOS category					0.52					0.007	0.86
≤ 3 days	11 (19%)	2 (8%)	5 (29%)	4 (25%)		9 (17%)	3 (12%)	2 (10%)	4 (57%)		
4–7 days	24 (42%)	12 (50%)	6 (35%)	6 (38%)		20 (38%)	8 (32%)	12 (57%)	0 (0%)		
8–14 days	14 (25%)	5 (21%)	5 (29%)	4 (25%)		17 (32%)	8 (32%)	7 (33%)	2 (29%)		
≥ 15 days	8 (14%)	5 (21%)	1 (6%)	2 (13%)		7 (13%)	6 (24%)	0 (0%)	1 (14%)		
Enteral feeding during admission^d^	26 (46%)	10 (41%)	9 (53%)	7 (44%)	0.76	31 (58%)	17 (68%)	11 (52%)	3 (43%)	0.38	0.18
Diagnostic endoscopic evaluation^e^	8 (14%)	2 (8%)	5 (29%)	1 (6%)	0.09	8 (15%)	4 (16%)	4 (19%)	0 (0%)	0.67	0.87
Restrictive eating disorder protocol	9 (16%)	9 (38%)	0 (0%)	0 (0%)	< 0.001	1 (2%)	1 (4%)	0 (0%)	0 (0%)	0.99	0.02
Psychiatry/Psychology consult	53 (93%)	24 (100%)	14 (82%)	15 (94%)	0.09	53 (100%)	25 (100%)	21 (100%)	7 (100%)	0.99	0.05
Nutrition consult	56 (98%)	24 (100%)	17 (100%)	15 (94%)	0.27	53 (100%)	25 (100%)	21 (100%)	7 (100%)	0.99	0.33
Social work consult	25 (44%)	4 (17%)	12 (71%)	9 (56%)	0.001	34 (64%)	12 (48%)	17 (81%)	5 (71%)	0.05	0.03
Readmission within 30 days^f^	5 (8%)	2 (8%)	1 (6%)	2 (13%)	0.79	3 (6%)	0 (0%)	2 (10%)	1 (14%)	0.16	0.53

*ARFID* Avoidant Restrictive Food Intake Disorder, *IQR* Interquartile Range, *AM* Adolescent Medicine, *GI* Gastroenterology, *GP* General Pediatrics, *BMI* Body Mass Index

^a^p-value testing for difference by admitting service within each period. Admitting and discharging service were the same

^b^p-value testing for difference overall between pre and post-ICP periods

^c^Between admit and discharge

^d^ Identified by nursing flowsheet entry for enteral formula intake

^e^Abnormal histopathology was identified for n = 5 (63%) patients in the Pre-ICP period and n = 7 (87%) Post-ICP period. In the pre ICP period, n = 4 esophagitis ranging from mild to moderate active esophagitis, 1 duodenal mucosa with focal, borderline increased intraepithelial lymphocytes and intact villous architecture, and 1 chronic inactive gastritis with Helicobacter organisms. In the post-ICP period, n = 1 neutrophilic esophagitis and chronic active gastritis related to indwelling NG tube, n = 1 superficial localized non-invasive candida in proximal esophagus, n = 3 esophagitis ranging from mild to moderate active esophagitis, n = 1 chronic inactive gastritis with Helicobacter organisms, n = 1 mild active distal colitis

^f^Excludes psych readmissions

### Aim 2: Development of a standardized ICP for treatment of patients with ARFID

In response to such variable care practices by service, the ICP was developed with collaboration of our multidisciplinary teams to standardize care implementation. Patients diagnosed with ARFID and with one of the following criteria were treated using the ARFID ICP: (1) Food and/or liquid refusal ≥ 24 h not explained by another medical or psychiatric condition; (2) Severe malnutrition as defined by ASPEN criteria [11] for BMI and/or acute weight loss; (3) Severe micronutrient deficiencies with risk of clinical impairment; and/or (4) Markedly poor intake of solid and/or liquids placing patient at risk for malnutrition and/or micronutrient deficiencies, refractory to outpatient management. Exclusion Criteria included: (1) Age < 3 years or > 21 years; and/or (2) Feeding/Eating Disorder other than ARFID. Care recommendations were incorporated into an order set embedded in the EMR to improve clinical compliance. A quality improvement database was created to examine the impact of the ICP over time with approval by our Institutional Review Board. The ICP was introduced and implemented for clinical care on January 1, 2018.

The ICP model of care followed specific principles (See Table 4) and was conducted in a step-wise fashion:

*STEP 1: Observation/Data Collection Period*: During the first 24–48 hours of inpatient admission, the patient is encouraged to eat *ad lib* and the patient’s dietary intake is recorded and analyzed for quality and quantity of macro and micronutrients. Nursing and clinical assistant staff observe mealtime behaviors both with and without the caregiver. Underlying co-morbid medical and psychiatric contributors and sequelae related to ARFID are identified.

*STEP 2: Care Plan Formulation and Implementation of Medical Care*: After the initial observation period, the multi-disciplinary team meets with the patient and caregivers to determine optimal methods to nutritionally stabilize and support the patient. Malnutrition, micronutrient deficiencies, and psychosocial impairment concerns are identified and addressed. Goals of the care plan include optimizing nutritional intake, developing mealtime structure to optimize the patient’s appetite and natural desire to eat, and support of desired behaviors to allow for graduated sustainable achievements.

*STEP 3: Family Education/Implementation of Care Plan and Discharge Planning*: As the hospitalization continues, the multi-disciplinary team works to empower the caregivers to implement the feeding plan to the best of their abilities through team member modeling and engagement in supportive non-accommodative behaviors. Behavioral parent training, psychoeducation regarding ARFID, and nutrition education are provided. Discharge planning is discussed in the framework of the patient’s projected needs and the caregiver’s competency with the intent of providing medical stabilization and positioning the family successfully to continue treatment via outpatient care.

**Table 4:** Descriptive principles and strategies of ARFID inpatient clinical pathway

Address underlying triggers and co-morbidities associated with ARFID	1. Medical/Psychiatric evaluation/care for underlying conditions contributing to development/persistence of feeding refusal
	a. Common medical disorders include Eosinophilic Gastrointestinal Disorders, Celiac Disease, Inflammatory Bowel Disease, GERD, Constipation, Disorders of Gut Brain Interaction
	b. Common psychiatric disorders include Anxiety, Depression, Obsessive Compulsive Disorder, Attention Deficit Hyperactivity Disorder, Autism Spectrum Disorder
	c. Evaluation for restrictive eating disorder: EDY-**Q** [21] and NIAS [22] surveys given to appropriate aged patients and caregiver
	2. Psychiatry consultation service (PCS)
	a. Psychotherapeutic evidence-based interventions for contributory disorders
Assess, address and support ingestion of adequate nutrition	1. Observe meals
	2. Evaluate patient’s nutritional status: Weight/Height/BMI, Diet composition of macro and micronutrients, micronutrient status [11, 13, 14]
	a. Nutritional rehabilitation:
	i. Correction of micronutrient deficiencies with supplements
	ii. Individualized determination of oral versus enteral supplemental nutrition needs (decision about need for supplemental enteral nutrition is based on degree of nutritional insufficiency and patient’s motivation and ability to implement oral nutritional restoration)
	b. Examine risk of re-feeding syndrome [23]
	i. Provide supplemental phosphorus and thiamine and monitor chemistries as indicated
Create structure for the patient and family around feeding to optimize appetite and awareness/importance of hunger and thirst sensation	1. Meals (referred to as “exposures”) occur daily at pre-determined times supervised by nursing and primary home caretaker(s) to support predictability
	a. 3–5 meal exposures daily. Up to 3 meals and 2 snacks (e.g., 30 min for meals, 20 min for snacks) and then tray is simply removed following exposure
	b. Mild verbal praise for meal completion and no additional engagement, cajoling, or otherwise for non-completion
	c. Patient should be sitting upright in a chair at table and can be engaging in passive distraction (e.g., conversation, puzzle, playing with fidget toy) if helpful
	2. Avoid grazing on foods/liquid between meals and snack times: If hunger between exposures, inform when the next meal/snack exposure will happen
	3. Develop/write a visible daily schedule to help patient expect/predict mealtimes and otherwise support behavioral activation (e.g., build in time for relaxation, coping, pleasant activities)
	4. Consider Cyproheptadine to improve appetite [24]. Hunger stimulation when hunger drive isn’t a concern could theoretically worsen meal time dysregulation. Side effect profile of cyproheptadine should be considered [25]
Creation of successful exposure therapy and meal plan	1. Create diet plan with patient/family commensurate with patient’s intake. Develop hierarchy of avoided foods and/or eating situations. Patient rates top 5 foods willing to eat, with modification suggestions supported by dietician, to optimize nutrient value of each food offered in protocol
	2. Initial exposure consists of small volumes (based on baseline data obtained in the observation period) to allow for 100% successful completion (e.g., one bite, a quarter of a waffle). Once patient is able to complete 100% of a given exposure, advance in small increment the next meal; may gradually increase incremental meal adjustments as subsequent successes are demonstrated
	3. Implement reinforcement system as appropriate (e.g., verbal praise, stickers, token reward system) for successful completion of exposures as presented
	4. Goal is to gently encourage progression with increased oral intake (and decrease in NG tube feeds as medically appropriate)
	5. As volume of PO intake becomes adequate, recommend transitioning to increasing diversity of PO intake over time. Skills are introduced inpatient with goal for expansion as outpatient
Address underlying anxiety with patient and family (when indicated)	1. Identify triggers of any contributing anxiety (eg; contamination, emesis, choking) or if patient only has disinterest in eating
	2. Psychoeducation to patient and caregiver(s) on the physiology of anxiety, role of avoidance in maintaining fear, and rationale for exposure-based treatment [26]
	3. Encourage relaxation strategies before, during, and after exposures (e.g., diaphragmatic breathing, progressive muscle relaxation, guided imagery)
	4. Avoid using language that might increase anxiety during exposures (e.g., hurry up, do this for me)
	5. If flooding of anxiety manifests- Consult with psychiatry regarding possible use of antianxiety medications, as indicated [27]
Educate and provide plan for caregiver role	1. Psychoeducation to decrease caregiver guilt and encourage sense of caregiver efficacy in supporting meal completion and treatment. Psychoeducation of ARFID [28–30]
	2. Patient–Caregiver Interaction
	a. Avoid pressure to finish meal, criticizing, engaging in emotionally charged topics, engaging in power struggles (e.g., Avoid “you have to finish it”)
	b. Limit responses to feeding avoidance, distraction, or complaint behaviors
	c. Keep the plate in front of patient for the duration of scheduled mealtime. At the end of the meal/snack time, remove plates without commenting on the amount of food left
	d. Consider reinforcement as appropriate (e.g., caregiver labeled praise, preferred activity)
	3. Use controlled choice by offering two options in order to provide a sense of agency while obtaining the same objective (e.g., offer a snack by asking the patient if she would prefer a goldfish snack or potato chips)
Optimize discharge planning to allow for progressive recovery	1. Consideration is placed on the needs of the patient and caretaker’s ability to provide supportive care and recovery
	a. Options for discharge may include transition to residential or intensive outpatient programs to address the eating disorder, comorbidities such as anxiety or depression or follow up in the multidisciplinary outpatient ARFID program
	b. Communication with primary care provider, school and family’s local providers is essential to successful transition to the outpatient setting

*ARFID* Avoidant Restrictive Food Intake Disorder, *GERD* Gastroesophageal Reflux Disease, *BMI* Body Mass Index, *EDY-Q* Eating Disturbance in Youth Questionnaire, *NIAS* Nine Item Avoidant Restrictive Food Intake Disorder Survey

### Aim 3: Comparison of pre- v. post-ICP implementation clinical outcomes and utilization

After ICP implementation, the variability by service in use of the restrictive eating disorder protocol and consulting services was attenuated (Table 3). Only one patient (4%) on the AM service in the post-ICP period was initially started on the restrictive eating disorder protocol compared to 9 (38%) in the pre-ICP period (p = 0.02). The one patient started on the restrictive eating disorder protocol was placed on the ARFID ICP during their hospital stay. All patients in the post-ICP period received both psychiatry/psychology and nutrition consults compared to 93% (p = 0.05) and 98% (p = 0.33) pre-ICP respectively. In the post-ICP period, there was still some variation in social work consults, ranging from 48 to 81% by service (p = 0.05). The lowest utilizer in the pre-ICP period, the AM service, increased from consulting social work 17% of the pre-ICP time to 48% of the post-ICP time with a significant overall change in social work consults with implementation of the ICP (p = 0.03). Length of stay declined from 7 days on average pre-ICP to 6.2 days on average post-ICP though this decline was not significant. Readmission rates remained low and were unchanged post-ICP implementation.

## Discussion

We demonstrate variability in care by admitting service for patients with ARFID prior to the development of a standardized ICP. The AM service commonly used a feeding protocol designed for patients with anorexia nervosa before the development of the ARFID ICP. With implementation of the ICP, overall utilization of consult services increased, particularly social work, nutrition and psychiatry and nearly eliminated the use of the restrictive eating disorder protocol. There was no worsening of LOS with the ICP and readmission rates remained low.

The publication of the DSM-5 in 2013 [1], marked the formalization of the ARFID diagnosis, In 2015–2017, the management of children with ARFID was without published guidelines. The inpatients in our study were clinically complex with nearly three-quarters exhibiting co-morbid anxiety and/or depression and over 60% defined as moderate or severely malnourished [11]. With the high complexity of needs among our inpatient cohort, the approach to care by the three medical services were similar in many ways, including frequent consultation of nutrition and psychiatry/psychology consultation service. However, variability both between and within the different medical services remained and may have impacted care.

The noted lack of standardized recommendations for care of patients with ARFID led our medical teams to rely on care approaches familiar to their expertise. Similar to studies from other eating disorder and ARFID-focused programs, [8, 12] almost half of the patients in our study initiated enteral tube feedings. The AM service, which commonly cared for patients with anorexia nervosa, employed the existing restrictive eating disorder protocol in approximately one-third of their admitted patients with ARFID prior to initiation of the ICP. The restrictive eating disorder protocol involved up to thrice daily placement and removal of a nasogastric tube designed to incentivize oral intake of meals. Anecdotally, we noted escalation in anxiety and feeding refusal with use of this protocol among some of our patients. Utilization of comparable restrictive eating disorder protocols is similarly employed among other adolescent medicine eating disorder programs across the country [8]. Guss et al. found that 55% of programs reported using a protocol developed for patients with anorexia nervosa while the remainder of the programs reported no guiding protocol [9].

Given variation in treatment approaches, we came together across admitting services and disciplines to standardize our approach. Examining patient care through the lens of the bio-psychosocial model, we created a standardized inpatient clinical pathway (ICP). Children and adolescents with ARFID often present with malnutrition and micronutrient deficiencies, and so the primary focus was on nutritional rehabilitation, first through increasing volume while later supporting expanding variety [3, 13, 14]. Recognizing that caregiver accommodation can deter progress, we provided opportunities for the patient to be fed with and without familial involvement. This served as a diagnostic opportunity (e.g., evidence of inadvertent behavioral reinforcement interfering with feeding) as well as opportunities for parent coaching [15, 16]. While our ICP addresses possible underlying medical disorders and assesses and manages nutritional sequelae of malnutrition, including risk of refeeding syndrome, it can simultaneously work to address psychosocial barriers to progress including psychiatric disorders and manifestations of parental accommodation. The ICP can allow for identification and generalized support for addressing anxiety, which is prevalent among individuals with ARFID [5] as demonstrated in our cohort. Successful implementation of behavioral strategies, in the children and adolescents, can be fostered by identifying and addressing caregiver accommodation [15, 16]. Once a framework has been implemented to optimize appetite and caregiver support, the ICP enables each meal to be an opportunity for trial and practice of food exposure and expansion, as have been described by a number of pilot studies [17, 18]. The ICP provides caregiver education in passive and active ways with modeling, guidance, and practice, enabling opportunities to better establish and maintain healthy parent child boundaries, utilizing healthy meals, and thereby optimizing the opportunity for success upon hospital discharge.

Our ICP was intended to provide guidance regarding the use of enteral tubes and implementation of the existing restrictive eating disorder protocol in patients with ARFID. It is possible that placement and removal of enteral tubes with each meal may further exacerbate anxiety in patients with ARFID and worsen avoidance and resistance to eating [19]. At times, nutritional rehabilitation with enteral tube is definitively indicated; however, we have learned that thoughtful implementation is needed instead of routine and/or repeated use [19]. In the ICP, education by way of possible use of enteral tube feeding supplementation is discussed early in the admission, with the patient and their family and the multidisciplinary team, with the goal of thoughtful implementation, when indicated, in the setting of nutritional benefits and/or as a supportive element for the patient.

Following implementation of the ICP, we examined a similar cohort of patients admitted for the treatment of ARFID. We chose to limit our examination to correspond to the initiation of the ICP in 2018 to just before declaration of the national emergency for COVID-19 pandemic in March 2020, so as not to create an additional modifier in patient care. The most significant was the change in the use of the protocol developed for restrictive eating disorders among patients treated pre- and post-ICP implementation. Among patients admitted to AM, over a third of patients during the pre-ICP period compared to only 4% post-ICP were placed on the restrictive eating disorder protocol compared with no patients on the GI or GP services. Furthermore, psychosocial consultations exhibited a trend toward less variability. Utilization of psychology and social work consults were both improved after ICP implementation and there was less variability by service in the post-ICP period. However, there were significant differences between services with regard to social work consultations in the post-ICP period; there were fewer social work consults in the AM service compared to the GI and GP services, signaling a need for improved standardization in this domain and better access to social work services. Consistent features across admitting services in pre- and post-ICP cohorts included frequent nutrition (94–100%) consultation. Among both cohorts of patients, enteral tube feedings were initiated in roughly half of patients without a noted change in BMI or LOS during the hospitalization.

This study has several strengths and multiple limitations. Our chart review included a review by 3 trained reviewers, allowing for consistency in patient selection to confirm admitting diagnosis of ARFID after querying admitting and discharge notes. Patients with an ambiguous diagnosis of an active underlying medical condition, at the time of admission, and ARFID were excluded from the study, as the etiology of feeding refusal was uncertain. This exclusion may explain, in part, the underrepresentation of underlying gastrointestinal disorders among our cohort compared to the literature [20]. It is also possible that the retrospective methodology may have missed some patients admitted with ARFID. Additional factors, such as severity of co-morbid disorders, the family’s ability to provide supportive care as an outpatient, provider bias, or other factors were not measured and may explain the team’s decision of whether to employ enteral tube feedings, diagnostic procedures and/or the existing restrictive eating disorder protocol. The relatively small cohort of patients examined likely resulted in low statistical power to detect a difference by service. Furthermore, the current study examined early changes after implementation of the ICP with a relatively limited number of patients. Additional research on the longer-term influence of the ICP will be beneficial to understand the longitudinal impact on health outcomes, cost-effectiveness, provider adherence to the ICP components, and perspectives of the health care providers and those receiving care. The impact, over time, of the ICP to inform the care of other eating disorders should be considered.

## Conclusion

We have reported on the variation in care in the management of ARFID, shortly after the classification of the new diagnosis for ARFID [1], and the potential impact of standardizing care with a clinical algorithm. We have learned that feeding refusal in the context of the ARFID diagnosis often presents with nutritional and medical sequelae along with psychiatric and psychosocial challenges. In addition, there has been an appreciation over time, of the differing etiologic underpinnings of restrictive eating disorders and ARFID [29, 30]. The need for a standardized approach to care directed at patients with ARFID informed our decision to create an inpatient clinical pathway centering the experience of the patient within the framework of more comprehensive biopsychosocial involvement in a care model.

## Data Availability

We are currently unable to share data.

## Abbreviations

ARFID: Avoidant restrictive food intake disorder
ICP: Inpatient clinical pathway
GI: Gastroenterology
GP: General pediatrics
AM: Adolescent medicine
BCH: Boston Children’s Hospital
QI: Quality improvement
PCS: Psychiatry/psychology consultation service
EMR: Electronic medical record
LOS: Length of stay
BMI: Body mass index
IQR: Interquartile range
