# Correction: Variation in care for inpatients with avoidant restrictive food intake disorder leads to development of a novel inpatient clinical pathway to standardize care

**DOI:** 10.1186/s40337-024-01035-7

**Published:** 2024-06-07

**Authors:** Elana M. Bern, Carly E. Milliren, Kevin K. Tsang, Lisa A. Mancini, Julia K. Carmody, Marina G. Gearhart, Olivia Eldredge, Chase Samsel, McGreggor Crowley, Tracy K. Richmond

**Affiliations:** 1 Division of Gastroenterology, Hepatology and Nutrition, Boston Children’s Hospital, 333 Longwood Avenue, 5th Floor, Boston, MA 02115 USA; 2 Department of Pediatrics, Harvard Medical School, Boston, MA USA; 3 Department of Psychiatry and Behavioral Sciences, Boston Children’s Hospital, Boston, MA USA; 4 Department of Psychiatry, Harvard Medical School, Boston, MA USA; 5 Institutional Centers for Clinical and Translational Research, Boston Children’s Hospital, Boston, MA USA; 6 Division of Adolescent/Young Adult Medicine, Boston Children’s Hospital, Boston, MA USA

**Correction to: Bern et al. Journal of Eating Disorders (2024) 12:66**

10.1186/s40337-024-01018-8

Due to typesetting error, the “Plain English Summary” section in the Original Article was incorrect. Some sentences were cut as follows:

There is limited evidence to guide management of children and adolescents with Avoidant Restrictive Food Intake Disorder (ARFID) admitted for me ARFID, which led to the development of a multidisciplinary standardized inpatient clinical pathway (ICP). The ICP centers the experience of the patient and family with an em increased standardized care for inpatients with ARFID with variation in care reduced: There were improvements in the use of psychiatry/psychology and social work consultin protocol. Future research is needed to better understand the impact of the inpatient clinical pathway to improve care over time.

The correct Plain English summary is as follows:

There is limited evidence to guide management of children and adolescents with Avoidant Restrictive Food Intake Disorder (ARFID) admitted for medical stabilization. The study describes the variation in inpatient care for ARFID, which led to the development of a multidisciplinary standardized inpatient clinical pathway (ICP). The ICP centers the experience of the patient and family with an emphasis on biopsychosocial support. Implementation of the ICP increased standardized care for inpatients with ARFID with variation in care reduced: There were improvements in the use of psychiatry/psychology and social work consulting services and a reduction in the use of the restrictive eating disorder protocol. Future research is needed to better understand the impact of the inpatient clinical pathway to improve care over time.

The original article has been corrected.

